# Auxetic Closed Cell Nylon Foams Produced by Vacuum and Mechanical Compression (VMC)

**DOI:** 10.1002/marc.202400274

**Published:** 2024-11-30

**Authors:** Xiao Yuan Chen, Denis Rodrigue

**Affiliations:** ^1^ Department of Chemical Engineering Université Laval Quebec G1V0A6 Canada

**Keywords:** auxetic materials, cellular structure, foam, mechanical properties, nylon

## Abstract

This research presents a method to convert a conventional Nylon closed cell foam, with a density of 48 kg m^−^^3^, into auxetic metamaterials with densities ranging between 68 and 138 kg m^−^^3^. The samples are produced by applying vacuum and mechanical compression techniques. The study reports on the effect of processing parameters such as vacuum time, temperature, and mechanical pressure. Under optimized conditions, the resulting auxetic foams exhibit a tensile Poisson's ratio as low as −0.86, while the minimum compressive Poisson's ratio for the same sample reached −0.16. Based on morphological analyses via scanning electron microscopy (SEM), structural changes induced by the treatment are determined to relate with tensile and compressive properties, including modulus and strength, as well as the analysis of the stress level for different strains. The characterizations also include differential scanning calorimetry (DSC) and dynamic mechanical analysis (DMA) for the Nylon foam before and after conversion. These findings underscore the potential applications of these auxetic foams in sports and military protective gears, as well as energy dissipation systems.

## Graphical abstract

Closed‐cell Nylon foams are converted into auxetic metamaterials with densities between 68 and 138 kg m^−^^3^. The samples are produced via different vacuum times, temperature, and mechanical pressure. The best foam has a tensile Poisson ratio of −0.86 and a compression Poisson ratio of −0.16. These foams have potential applications in sports and military protective gears, as well as energy dissipation systems.

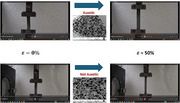

## 1 Introduction

Nylon foam (NF) is a lightweight and versatile material featuring very high‐temperature resistance compared to other polymer foams available on the market. It is able to maintain its useful properties for temperatures as high as 204 °C depending on the grade.^[^ ^1^ ^]^ Compared to other polymer foams, such as polystyrene foam (beginning to soften ≈100 °C, and melt point at 240–260 °C),^[^ ^2^ ^]^ polypropylene (PP) foam (≈140 °C)^[^ ^3^ ^]^ and polyethylene (PE) foam (≈80 °C for LDPE, and 120–140 °C for HDPE depending on the grade),^[^ ^4^ ^]^ Nylon foam has a higher melting point (≈205–230 °C depending on the grade). Nylon foam has excellent durability and resistance, as it can withstand frequent and heavy loads without warping or degradation,^[^ ^1^ ^]^ outperforming several other foams. Compared to traditional polyurethane (PUR) and polyethylene (PE) foams, Nylon foams offer superior structural integrity and resilience. While PUR foams are known for their flexibility and softness, they tend to degrade more rapidly under continuous heavy loads. On the other hand, PE foams provide good cushioning but lack the same resistance to compression and wear over time. NF is also non‐toxic, odorless, buoyant, and non‐absorbent with excellent recovery from repeated impacts and resistance to a wide range of chemicals, oils, and fuels. Additionally, it can be easily shaped, cut, and bonded to other materials, making it suitable for various applications. Potential industries benefiting from NF include automotive, seals, gaskets, personal protection, thermal insulation, heat shields, and energy absorbers. NF is notably incorporated into sporting equipment, such as helmets, pads, and protective gear, to absorb impact and reduce the risk of injury.^[^ ^5^ ^]^

Auxetic materials, with their negative Poisson's ratio (NPR), exhibit a unique behavior as they expand laterally when stretched and contract when compressed. Over time, researchers have achieved the transformation of various polymer foams into auxetic foams with NPR: polyethylene (PE),^[^ ^6^, ^7^, ^8^, ^9^, ^10^ ^]^ polyether urethane (PU),^[^ ^11^, ^12^, ^13^, ^14^, ^15^, ^16^ ^]^ polypropylene (PP),^[^ ^3^ ^]^ polyvinyl chloride (PVC),^[^ ^8^ ^]^ and polyester (PES).^[^ ^17^ ^]^ NPR is a property associated with the structural design of materials, such as cellular structures, rather than an intrinsic attribute of the material itself. The unique morphology of these structures imparts distinct mechanical properties, such as improved elasticity and resistance to diverse forms of deformation or stress, especially compression, tension, and impact.

The main concept behind the production of auxetic foams from conventional ones involves converting the original honeycomb or polyhedral‐shaped cell morphology into a re‐entrant cellular structure.^[^ ^6^, ^7^, ^8^, ^9^, ^10^, ^11^, ^12^, ^13^, ^14^, ^15^, ^16^, ^17^ ^]^ This conversion can be accomplished through chemical, mechanical, physical, and thermal treatments, either individually or in combination. Typically, these processes involve compressing the foam, heating it above the polymer's softening point, and subsequently cooling it while maintaining compression to fix the structure generated.

In our previous studies, two distinct methods were proposed to produce auxetic foams from waste foams (upcycling). The first method involves a combination of solvent (ethanol) evaporation–condensation and mechanical compression (SECC),^[^ ^18^ ^]^ while the second imposes vacuum and mechanical compression (VMC).^[^ ^3^, ^4^ ^]^ The latter is used in this study to convert conventional Nylon foams into NPR materials. This method combines heat and pressure to soften and compress the foam to deform the cells, followed by cooling while maintaining pressure to stabilize the re‐entrant cellular structure generated. To the best of our knowledge, this is the first successful attempt to directly upcycle Nylon foams into auxetic metamaterials. Based on the samples produced, the effect of mechanical stress, heating, and vacuum time are determined to optimize the final foam structure. Tensile and compressive stress‐strain curves were measured to determine their respective Poisson's ratios. These results will help to improve our understanding of auxetic polymer foams in terms of production and properties in a broader context.

## 2 Results and Discussion

### 2.1 Physical Properties of the Nylon Foams

The initial density of the Nylon foam (N─O) is 48.0 kg m^−3^ and this serves as the baseline for all the samples detailed in **Table** **1**. Each sample is coded with a “N” for the foam type (Nylon), followed by “Tx” indicating the treatment temperature (190–205 °C), followed by a number indicating the duration of vacuum (1–36 h). Lastly, “Px” is the applied mechanical pressure (kPa). For instance, sample N‐T205‐24‐P5.0 refers to a Nylon 6 foam treated at 205 °C for 24 h under a mechanical pressure of 5 kPa. All the samples were produced under maximum oven vacuum conditions (−0.88 bar) as determined to be the optimal condition based on our previous investigations.^[^ ^2^, ^3^, ^4^ ^]^

**Table 1:** Physical properties of the original Nylon and auxetic foams.

Sample	Density [kg m^−3^]	Porosity [%]	OCP [%]
N─O	48.0 ± 2.1	95.9 ± 0.9	16.8 ± 0.7
N‐T190‐16‐P1.0	68.0 ± 5.3	94.2 ± 5.2	12.9 ± 1.0
N‐T200‐16‐P1.0	114.2 ± 4.5	90.2 ± 4.7	13.2 ± 0.5
N‐T205‐6‐P1.0	73.3 ± 4.7	93.7 ± 5.9	10.6 ± 0.7
N‐T205‐10‐P1.0	84.8 ± 6.9	92.7 ± 7.2	11.2 ± 0.9
N‐T205‐16‐P1.0	120.8 ± 4.8	89.6 ± 3.5	13.0 ± 0.5
N‐T205‐24‐P1.0	124.4 ± 4.7	89.3 ± 3.5	18.1 ± 0.7
N‐T205‐36‐P1.0	135.1 ± 6.3	88.3 ± 7.0	18.9 ± 0.9
N‐T205‐24‐P5.0	138.0 ± 5.4	88.1 ± 5.4	20.1 ± 0.8
N‐T205‐24‐P10.0	138.2 ± 4.8	88.1 ± 6.3	21.2 ± 0.7

Table 1 provides a comprehensive overview of the physical properties of the foams. The results show that the density of the auxetic foams increases with increasing temperature and vacuum exposure duration. As temperature increases, the foam cells become more flexible and deform under compression, resulting in higher density. Longer vacuum time removes more gas molecules from the foam, leading to higher compression and higher density. Initially, applying pressure compresses the foam cells, increasing density. However, after a certain point, more pressure does not significantly increase the density, reaching a plateau as samples N‐T205‐24‐P5.0 and N‐T205‐24‐P10.0 exhibit similar densities (138 kg m^−3^).

Conversely, porosity has a decreasing trend with increasing temperature, vacuum duration, and mechanical pressure. As higher temperatures compress more the foam cells, this decreases the volume of empty space (porosity) inside the foam. When pressure compresses the foam, it forces the gas molecules out, thus decreasing porosity. Extended vacuum exposure enhances the pressure difference between the inside and outside of the foam cells, collapsing them further and lowering porosity.

The open cell percentage (OCP) displays two distinct trends. At low density (below 120 kg/m^3^), the OCP of the auxetic foams is lower than that of the original Nylon foam (16.8%). At lower densities, increasing temperature causes the cells to deform without rupture, resulting in a low OCP. As density increases, the cell walls may rupture increasing OCP. Similar to temperature, pressure initially causes deformation, but higher pressures can break the cell walls increasing OCP. Over time, vacuum exposure can increase OCP as cells collapse and break, especially at higher densities.

**Figure 1:**
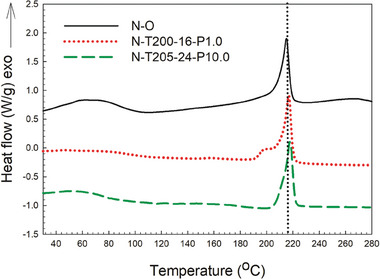
Typical DSC curves for the original Nylon and auxetic foams.

The DSC thermograms depicted in **Figure** **1** illustrate the thermal properties of both the initial Nylon foams and their counterparts after conversion. Upon comparing the results with the original foams (**Table** **2**), several observations can be made. The curve for the N─O foam exhibits a minor peak in the 40–70 °C range, potentially indicating the presence of contaminants or small molecules such as additives or volatiles. The softening point for the original Nylon foams begins ≈170–180 °C (DSC onset temperature), with a peak melting point ≈215 °C. In contrast, for both auxetic foams, the softening points are slightly delayed, starting at 190 and 200 °C respectively, while their melting points remain similar ≈217 °C.

**Table 2:** DSC results (main peak) of the original Nylon foam and auxetic foams.

Sample	*T_m_* [°C]	△*H_m_* [J/g]	*X_c_* [%]
Original Nylon foam (N─O)	215.2	83.4	34.6
Auxetic foam (N‐T200‐16‐P1.0)	217.0	72.7	30.1
Auxetic foam (N‐T205‐24‐P10.0)	217.6	50.0	20.8

A comparison of the auxetic foams with the original sample shows no significant difference between the melting points before and after conversion into auxetic foams, but a significant decrease is observed in their crystallinity. The original foam has a higher crystallinity (34.6%), which decreases to 30.1% and 20.8% after treatment. This suggests that the VCM process applies both thermal and mechanical energies to the original foam, resulting in the formation of the re‐entrant morphology characteristic of auxetic materials. However, this process also leads to a reduction in the long‐range order within the foam structure, resulting in lower crystallinity.

The conversion of Nylon foams changes their structure and crystallinity without significantly altering their melting points, because the conversion, involving temperature, pressure, or vacuum, affects how the polymer chains are arranged, while the foam's chemical structure (Nylon's polymer backbone) is unchanged;, i.e., the conversion reorganizes the internal structure of the foam, which exhibit a re‐entrant or folded structure, allowing them to behave in this unique way (See section 3–2‐1 SEM photos).

### 2.2 Auxetic foams

#### 2.2.1 Morphology

**Figure 2:**
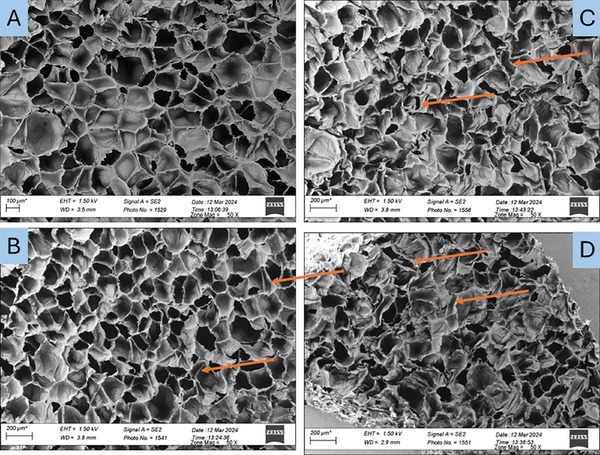
Morphology (cross‐section) of: A) original foam (N─O) as well as selected auxetic foams: B) N‐T200‐16‐P1.0, C) N‐T205‐24‐P1.0, and D) N‐T205‐24‐P10.0.

The SEM micrographs provided in **Figure** **2** offer an overview of the structural changes undergone by the Nylon foams and associated with their modification to determine the effect of the conversion process. Figure 2A shows that the original foam exhibits a homogeneous distribution of fine cells throughout the sample. The cellular structure mainly consists of closed‐cell (global polyhedral morphology) with an average cell diameter of 172 ± 51 µm. After modification, significant changes in cell size and foam morphology are observed. In Figure 2B, most cells are smaller than in the original foam, with an average diameter of 114 ± 89 µm and an elliptical shape. This reduction (34%) is likely due to the absence of a re‐entrant structure at 200 °C. But Figure 2C,D clearly show a re‐entrant structure as most of the cells collapsed inward. In this case, the quantification of the cell size becomes challenging due to the complex shape/geometry resulting from the re‐entrant structure. Interestingly, despite being produced under different mechanical pressures, the foams exhibit similar structures, highlighting the robustness and reproducibility of the conversion process.

#### 2.2.2 Poisson's ratio

The Poisson's ratio was determined through both tensile and compressive tests. The results show that the original Nylon foam exhibits a semi‐rigid and isotropic behavior with a density of 48 kg m^−3^. Under tension, the average Poisson's ratio is 0.30 which corresponds to a tensile elongation of up to 86%. Conversely, during compression, the average Poisson's ratio dropped significantly to 0.03 despite achieving a compressive strain of 83% (Figure 6). This difference underscores the foam's higher resistance to deformation under compression compared to tension and these findings are in line with a previous study investigating the tensile Poisson's ratio of polyamide 6 (PA 6) foams with densities of 30 and 50 kg m^−3^.^[^ ^22^ ^]^ According to their measurements, the tensile Poisson's ratios for these foams were 0.398 and 0.319 respectively, without data available for compressive strain.

To improve the post‐treatment (negative Poisson's ratio), an optimization of the processing conditions was carried out. During this process, the maximum vacuum level (─0.88 bar) was applied for all experiments. However, it was imperative to control the other parameters such as the temperature, vacuum duration, and mechanical pressure. The effect of each factor is presented next.

#### 2.2.3 Treatment Temperature

As previously stated, the temperature must be above the polymer's softening point, but below its melting point. Based on the DSC curves of the Nylon foam, the softening point is ≈185 °C (DSC onset temperature) with a melting point of 215 °C (DSC peak temperature). Consequently, we selected 190, 200, and 205 °C as the treatment temperature.

**Figure 3:**
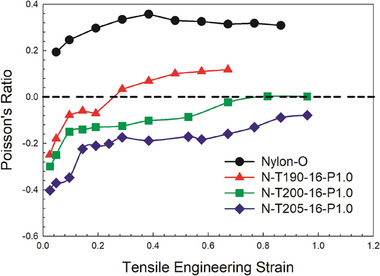
Poisson's ratio as a function of engineering strain (tension) for auxetic foams treated at different temperatures under vacuum (─0.88 bar) for 16 h.

**Figure** **3** illustrates the Poisson's ratio as a function of the tensile engineering strain across the treatment temperature range. These samples were produced under low mechanical pressure (1 kPa) to stabilize the samples (achieve a flat surface), coupled with 1 h of heating time and 16 h of vacuum exposure. Negative Poisson's ratios were observed for all the samples up to a deformation range of 70–100%. In particular, the NPR was more negative at 205 °C compared to other temperatures. Theoretically, higher temperatures make the foam softer leading to easier deformation of the cell walls and the generation of a re‐entrant structure, thereby yielding a lower NPR. Moreover, the Poisson's ratios consistently increased with increasing engineering strain. Based on these insights, 205 °C was selected for subsequent steps as higher temperature made the foams too soft and it was not possible to generate good samples (almost complete collapse).

#### 2.2.4 Heating and Vacuum Time

**Figure 4:**
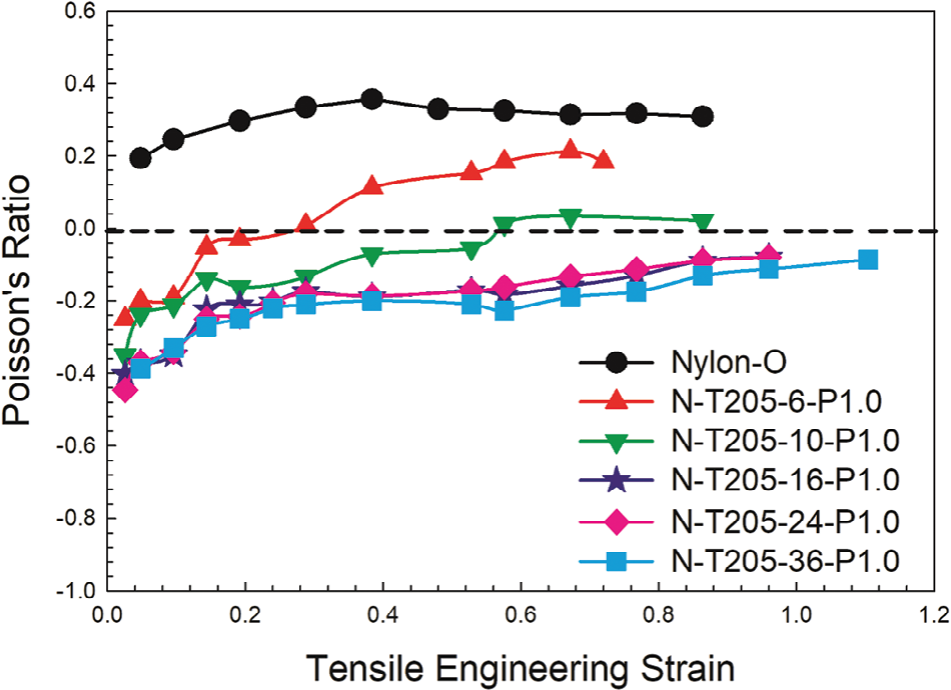
Poisson's ratio as a function of engineering strain (tension) for auxetic foams treated under vacuum (−0.88 bar) for different times at 205 °C.

**Figure** **4** illustrates the effect of vacuum time (6, 10, 16, 24, and 36 h). As the foam was exposed to vacuum, the pressure within the cells gradually equalizes with the external pressure. Upon returning the oven to ambient pressure, the external air pressure surpasses that within the foam cells. This increased pressure compresses the foam, inducing rapid shrinkage and partial collapse of cell walls, thereby generating a re‐entrant structure. Subsequently, upon cooling to room temperature, the deformed cell structure becomes locked in place. Thus, the main driving force behind this phenomenon is the pressure difference between the exterior and interior of the cells, i.e., the pressure difference across the cell walls. In theory, longer vacuum periods imply that the force generating the re‐entrant structure is more easily reached (achieve equilibrium).

A further analysis of the results in Figure 4 shows that the NPR values decrease as the vacuum time increases from 1 to 36 h and the minimum PR was ≈−0.4 as no significant difference was observed above 16 h. To be safe, 24 h was selected as the optimal duration (equilibrium) for the next steps. From our previous work on low‐density polyethylene (LDPE) foams, it was found that only 3 h of vacuum time was sufficient to achieve equilibrium.^[^ ^2^, ^3^, ^4^ ^]^ However, polypropylene (PP) and polystyrene (PS) foams needed 15 h of vacuum time.^[^ ^2^, ^3^ ^]^ This difference is attributed to the higher rigidity (modulus) of PP and Nylon compared to LDPE, necessitating extended vacuum time for Nylon foams to achieve equilibrium and fully convert into auxetic foams.

#### 2.2.5 Mechanical pressure

The idea behind applying mechanical pressure is to induce higher levels of deformation/stress, thus facilitating the formation of the re‐entrant structure. This condition also leads to higher foam density. However, excessive compression can lead to cell collapse, thereby compromising the integrity of the generated re‐entrant structure. This trend was also reported in our previous investigations.^[^ ^2^, ^3^, ^4^ ^]^

**Figure 5:**
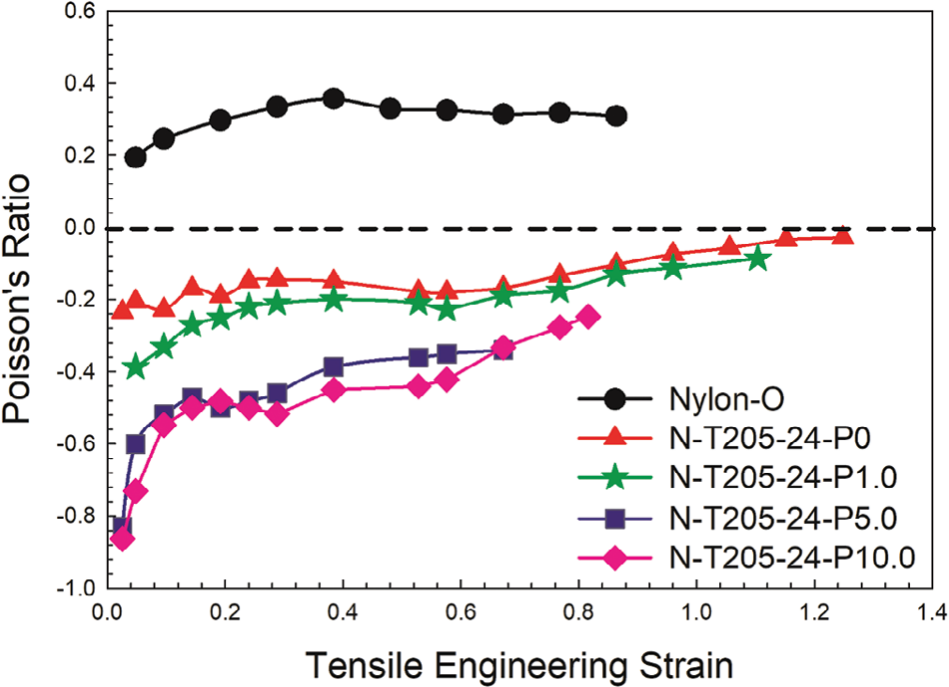
Poisson's ratio as a function of engineering strain (tension) for auxetic foams treated under different mechanical pressure at 205 °C for 24 h with vacuum (−0.88 bar).

To optimize the mechanical pressure for Nylon foams, four levels (0, 1, 5, and 10 kPa) were applied concurrently with the maximum vacuum (−0.88 bar) for 24 h at 205 °C. **Figure** **5** presents the Poisson's ratios as a function of the tensile engineering strain for the different mechanical pressures applied. A comparison between P0 and P1.0 shows similar trends, although a reduction in the minimum Poisson's ratio was observed. A similar trend is observed for P5.0 and P10.0 groups, but the curve for P10.0 is slightly lower than that of P5.0, although the difference might not be statistically significant. These results show that the minimum Poisson's ratios obtained are ≈−0.86.

For the auxetic foams presented in Figure 5, the Poisson's ratio was still negative up to 120% of tensile engineering strain. Although the Poisson's ratio increased with engineering strain, the curves did not display a linear dependence with axial strain. This behavior was also reported by Evans et al.^[^ ^23^ ^]^ and Lakes et al.,^[^ ^24^ ^]^ despite working with different materials (expanded polytetrafluoroethylene and mixed polyurethane‐polyester foam, respectively) each showing a distinct structure. This trend was also reported several times in the literature for NPR foams.^[^ ^2^, ^3^, ^4^, ^6^, ^7^, ^23^, ^24^, ^25^ ^]^ Unfortunately, no data on Nylon auxetic foams have been reported in the literature to compare.

**Figure 6:**
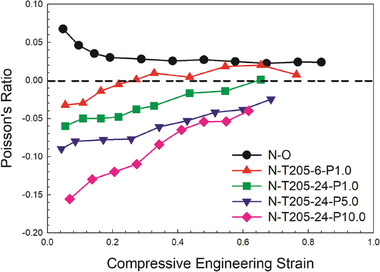
Poisson's ratio as a function of engineering strain (compression) for N─O and auxetic foams treated with different mechanical pressure at 205 °C with 6–24 h of vacuum (−0.88 bar).

To further enhance the analysis, **Figure** **6** presents the Poisson's ratio plotted as a function of the compressive engineering strain for auxetic foams treated under varying mechanical pressures (0–10.0 kPa). For comparison, N‐205‐6‐P0 reveals that a shorter vacuum time without mechanical pressure resulted in a less negative Poisson's ratio. Furthermore, the Poisson's ratio becomes positive above 25% of strain. It is clear that the NPR decreases as the mechanical pressure increases from 0 to 10.0 kPa, as expected. Theoretically, higher mechanical pressure exerts higher force on the samples, facilitating the generation of a re‐entrant structure and subsequent cell collapse, leading to higher foam densities (Table 1). In this case, the Poisson's ratio increases with compressive engineering strain and remains negative up to a strain of 60%. This is in agreement with the findings of Choi et al.^[^ ^24^ ^]^ and Lisieck et al.,^[^ ^25^ ^]^ despite using different materials as the former examined a mixture of closed and open‐cell polyurethane‐polyester foam with a density of 30 kg m^−3^, while the latter studied open‐cell PU foam with a density of 25.3 kg m^−3^. The nonlinearity observed in the compressive curves can be attributed to the complex relationships between the re‐entrant structure (cell geometry and size), imposed deformation, and resulting Poisson's ratio.

**Table** **3** reports on the minimum PR under tension and compression as a function of the compression ratio. Based on all the processing conditions investigated, the optimum was found to be a compression ratio of 2.88 for N‐T205‐24‐P10.0 yielding a minimum PR of −0.86 in tension and −0.16 in compression. As for other rigid plastic foams,^[^ ^26^ ^]^ the PR (absolute value) is lower in compression compared to tension.

**Table 3:** Properties of the auxetic samples based on Nylon foams.

Sample	Final density [kg m^−3^]	Compression ratio (ρ_f_/ρ_o_)	Minimum PR (tension)	Minimum PR (compression)
N‐O	48.0	1	0.30 (average)	0.03 (average)
N‐T205‐6‐P1.0	73.3	1.53	−0.24	−0.03
N‐T205‐24‐P1.0	124.4	2.59	−0.38	−0.06
N‐T205‐24‐P5.0	138.0	2.88	−0.83	−0.08
N‐T205‐24‐P10.0	138.2	2.88	−0.86	−0.16

#### 2.2.6 Mechanical Properties

##### Stress‐Strain Plots

**Figure 7:**
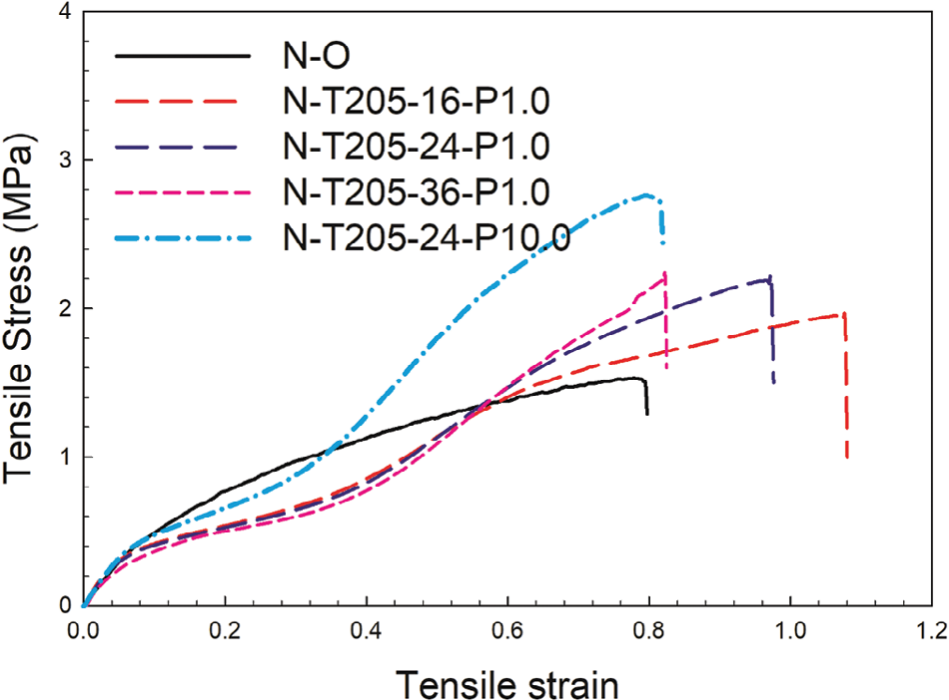
Tensile stress as a function of strain for the original and re‐entrant (auxetic) foams.

In order to complete the mechanical analysis of the auxetic foams produced, **Figure** **7** compares the stress‐strain curves in tension and compression before and after treatment. In Figure 7A, the stress‐strain curves for both the original and re‐entrant foams are compared in tension, before and after treatment. These curves illustrate the relationship between engineering stress and engineering strain during tensile testing. The corresponding mechanical properties, including tensile modulus, tensile strength, and strain at break, are summarized in **Table** **4**.

**Table 4:** Tensile properties of the Nylon auxetic foams produced.

Sample	Density [kg m^−3^]	Modulus [kPa]	Strength [MPa]	Strain at break [%]
N─O	48.0 ± 2.1	53.2 ± 8.0	1.34 ± 0.38	70.1 ± 17.1
N‐T205‐16‐P1.0	114.2 ± 4.5	59.1 ± 5.8	1.90 ± 0.22	107.7 ± 10.2
N‐T200‐24‐P1.0	124.4 ± 4.7	58.2 ± 4.6	2.19 ± 0.30	97.6 ± 9.5
N‐T205‐36‐P1.0	135.1 ± 6.3	50.1 ± 5.3	2.20 ± 0.23	82.5 ± 7.2
N‐T205‐24‐P10.0	138.2 ± 4.8	65.1 ± 5.9	2.75 ± 0.18	82.0 ± 6.9

The Young's modulus of N‐O is 53.2 kPa, which is similar to the values of the auxetic foams, except for N‐205‐24‐P10.0, which has the highest density. This similarity can be attributed to the higher final density of the treated samples. Additionally, tensile strength increases with increasing final foam density as more material is available to withstand the applied stresses. This relationship is directly proportional to the amount of material (density) present.^[^ ^27^, ^28^, ^29^ ^]^

Moreover, the elongation at break decreases with increasing density. Despite this trend, the auxetic foams exhibit substantially higher elongation at break (82–107%) compared to N─O (70%). This indicates that auxetic foams have superior ductility or ability to deform under tensile stress before failure.

Understanding these mechanical properties and the effect of density provides crucial insights into the behavior and performance of auxetic foams, which can be used to determine their applications in various engineering areas.

**Figure 8:**
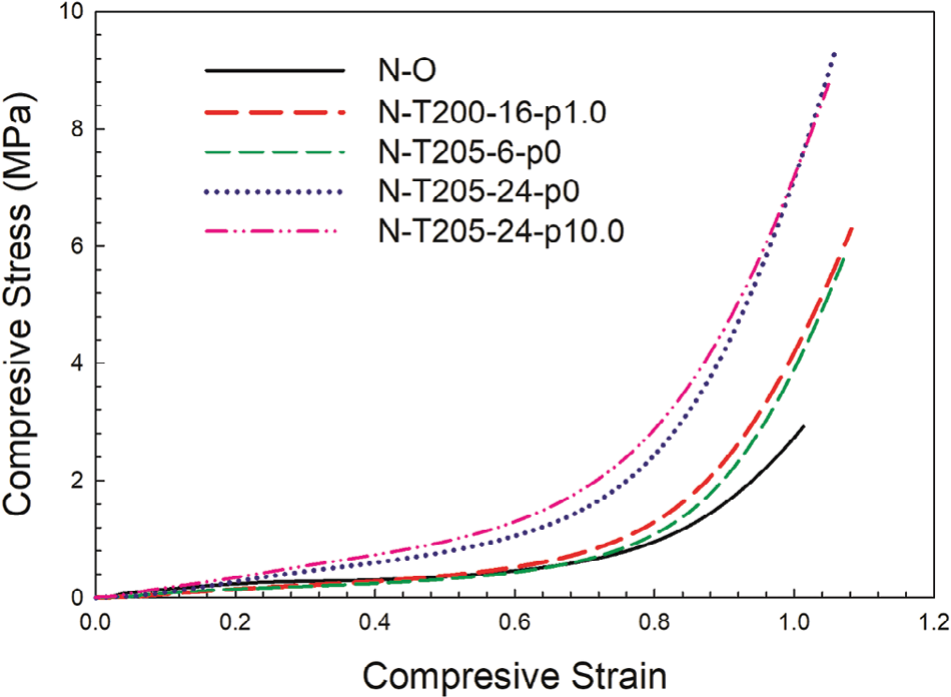
Compressive stress‐strain curves of the Nylon auxetic foams produced.

The compressive mechanical properties were also examined in terms of modulus, and stress at various strains (5%, 25%, 50%, and 100%), and the values are listed in **Table** **5**. Under compression, the modulus of the original foam (N─O) was higher than most of the auxetic foams, except for N‐T205‐24‐P10.0 despite having higher densities. The curve for N─O exhibits three regions: a linear elastic portion, a plateau, and then exponential growth. However the auxetic foams lack a definite plateau region and showed only exponential growth. At small deformations (≤5%), the stress and strain are proportional due to cell wall bending. However, the auxetic foams have lower force (resistance) at small deformations due to their cell walls being already bent. At higher deformations (e.g., 100%), the foams collapsed under compression with minimal stress increase, attributed to cell wall buckling. This lack of a plateau region is related to the cell ribs already being bent inward (re‐entrant structure) which are further bent rather than buckled. The stress at 100% strain also increases with the amount of material (density) present. For strains between 25% and 50%, the stress exhibits a similar trend for all the foams except for N─O. However, at strains above 60%, the stress increases similarly to tensile deformation: increasing with final foam density. Despite the variations, all the curves show similar trends **Figure** **8**.

**Table 5:** Compressive properties of the Nylon auxetic foams produced.

Samples	Density [kg m^−3^]	Compression			
		Modulus (kPa) [0–5%]	Stress at 25% strain [kPa]	Stress at 50% strain [kPa]	Stress at 100% Strain [MPa]
N─O	48.0 ± 2.1	890 ± 9.5	190 ± 2.0	360 ± 3.8	2.73 ± 0.03
N‐T205‐6‐P1.0	114.2 ± 4.5	406 ± 4.3	178 ± 1.9	316 ± 2.9	3.90 ± 0.05
N‐T200‐16‐P1.0	124.4 ± 4.7	332 ± 3.9	288 ± 3.4	373 ± 4.1	4.22 ± 0.06
N‐T205‐24‐P1.0	135.1 ± 6.3	553 ± 5.8	367 ± 3.1	780 ± 8.2	7.15 ± 0.11
N‐T205‐24‐P10.0	138.2 ± 4.8	1297 ± 10.6	443 ± 4.9	957 ± 10.1	7.20 ± 0.09

##### Dynamic Mechanical Analysis (DMA)

Dynamic Mechanical Analysis (DMA) is a versatile and sensitive technique enabling a thorough exploration of relaxation mechanisms in viscoelastic materials. One of the most common uses of DMA is to determine transition temperatures, where the maximum loss of applied energy is observed, typically appearing as a peak in the loss factor trace as a function of temperature.

**Figure 9:**
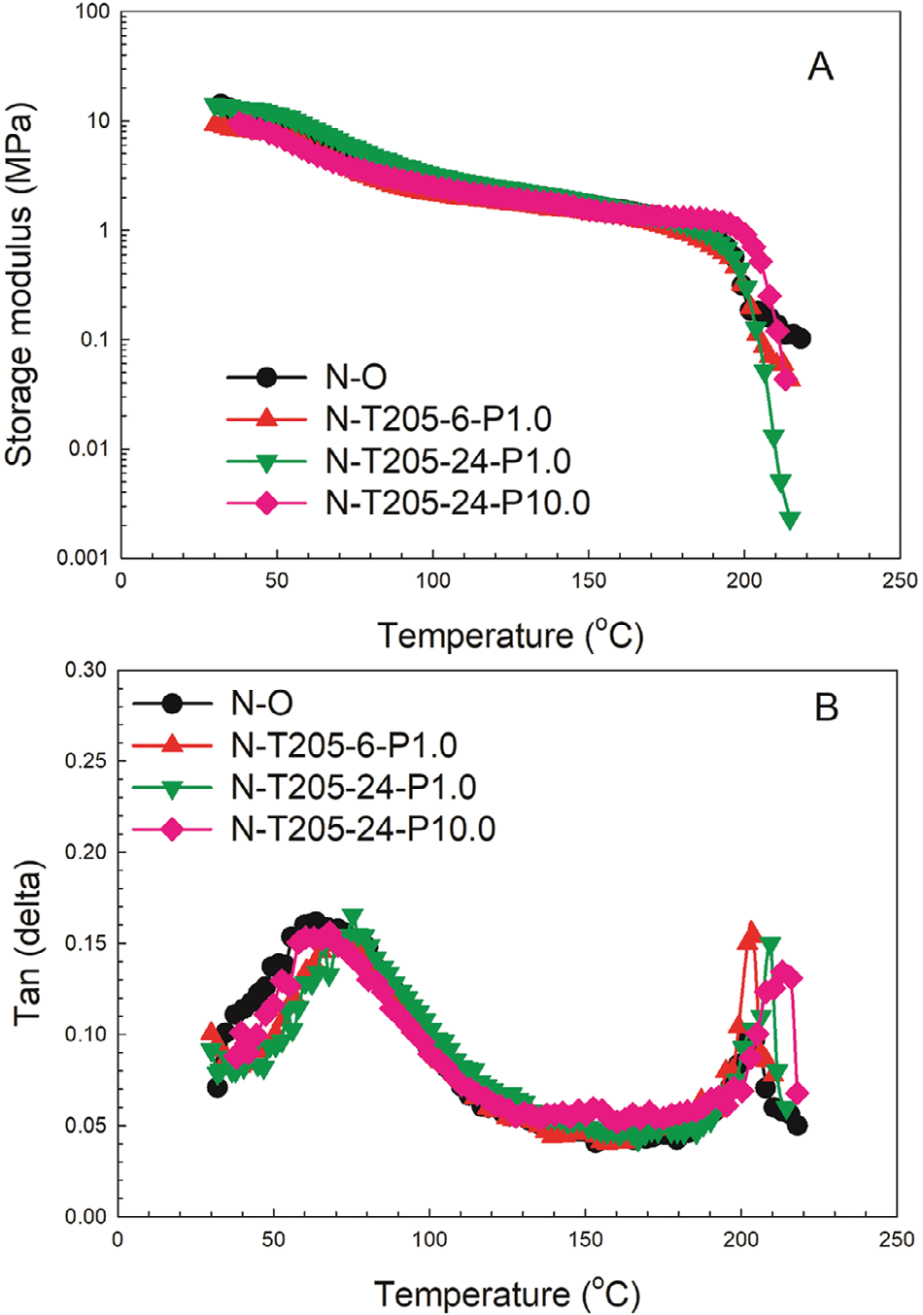
Storage modulus and Tan (δ) as a function of temperature for Nylon foams before and after treatment.

DMA can provide insights into temperature transitions by observing changes in the storage modulus or the Tan(δ) curve. A significant drop in the storage modulus or a peak in Tan(δ) often indicates these transitions. **Figure** **9** shows both effects:

1. The storage modulus curve (Figure 9A) shows a gradual decrease with temperature, which can indicate softening or the onset of a transition.
2. In the Tan(δ) curve (Figure 9B), peaks suggest the presence of phase transitions from the viscoelastic response.

The storage modulus represents the material's rigidity, which decreases with increasing temperature (Figure 9A), showing consistent trends across all tested materials. According to Figure 9B, the melting temperatures are 204, 204, 209, and 215 °C for N─O, N‐T205‐6‐P1.0, N‐T205‐24‐P1.0, and N‐T205‐24‐P10.0, respectively. A comparison between the original foam (N─O) and the treated auxetic foam with different vacuum times and mechanical pressure reveals that higher rigidity (higher density) correlates with higher melting points.

The glass transition temperatures (Tg) are between 64 and 78 °C for all samples. The re‐entrant structure results in a more compact and rigid configuration, leading to higher transition temperatures.

## 3 Conclusion

This study presented a novel approach to produce auxetic materials (68‐138 kg m^−3^) from a conventional Nylon foam (closed cell, 48 kg m^−3^) using a vacuum and mechanical compression method. Through systematic experimentation and analysis, the optimal treatment conditions were identified as: 24 h of vacuum (−0.88 bar) at 205 °C under 10 kPa of mechanical pressure leading to the successful conversion of a standard Nylon foam into auxetic metamaterials with negative Poisson's ratios.

The auxetic foams produced were shown to have superior mechanical properties compared to the original (non‐auxetic) sample. Under tension, the auxetic foams exhibited a minimum tensile Poisson's ratio of −0.86, while the values were only −0.16 under compression. Young's modulus (from 53.2 to 65.1 kPa), strength (from 1.34 to 2.70 MPa), and elongation (from 70 to 82%) were significantly improved under tension. At the same time, the compressive modulus (from 890 to 1296 kPa) and stress at 25% (from 190 to 443 kPa), 50% (from 360 to 957 kPa), and 100% (2.7 to 7.2 MPa) of strain were also improved.

The study provided valuable insights into the effect of various processing parameters on the final foams' physical, thermal, morphological, and mechanical properties. These findings contribute to our understanding of auxetic polymer foams in terms of production and properties. These results can open new possibilities for their application in diverse areas such as automotive, seals, gaskets, personal protection gears, functional thermal insulation, and energy absorption. Overall, this research laid the ground for further exploration and use of auxetic materials in engineering and material science fields.

## 4 Experimental Section

### Materials

In this study, Nylon 6 foam samples were purchased from McMaster‐Carr.^[^ ^19^ ^]^ An economical alternative to Viton fluoroelastomer foam, these semi‐rigid Nylon foam sheets offer good resistance to a wide range of chemicals. The foams have a closed‐cell structure limiting water, air, and other gases from being absorbed. These foams (30× 30 × 2.54 cm) had an initial density of 48 kg m^−3^. To perform the treatments, a vacuum oven (OVV‐400‐24‐120 Programmable Vacuum Oven, Cole‐Parmer, USA) was used.

### Methods

The Nylon foams underwent a series of treatments as follows. Initially, the material was cut into pieces of 10 cm × 10 cm × 2.54 cm using a doctor blade. Subsequently, each sample was placed inside a vacuum oven and subjected to heat for 1 h at temperatures ranging from 190 to 205 °C. This temperature range was selected to be close to the Nylon softening temperature (204 °C) but below its melting temperature (220 °C). Then, the oven underwent a vacuum process (maximum −0.88 bar) for durations between 1 and 36 h at temperatures between 190 and 205 °C, followed by a rapid return to ambient pressure through fast decompression. During this treatment, mechanical pressure ranging from 0 to 10 kPa was also applied to the sample using a metal block of varying weight. After completion of the treatment cycle, the sample was allowed to cool inside the oven for 1 to 2 h while still under mechanical pressure. The final volume of the foam produced during the vacuum and mechanical compression (VMC) process represents a balance between the foam's expansion due to the elevated temperature and the vacuum/mechanical stress (weight) applied.^[^ ^2^, ^3^, ^4^ ^]^

### Characterization

### Characterization—Characterization of the Nylon Foams

The bulk density of the foams (*ρ_f_*) was obtained from the ratio between its weight (*W_f_*) and its volume (*V_f_*) calculated by measuring the length (*L*), width (*W*), and thickness (*l*) with a digital caliper (Mastercraft, USA). Each dimension was taken at four locations and averaged to calculate:

(1) $$\rho_{f}=\frac{W_{f}}{V_{f}}=\frac{W_{f}}{L\;W\;l}$$

The porosity (*P*) was calculated as:

(2) $$P=\frac{\rho_{l}-\rho_{f}}{\rho_{l}-\rho_{a}}\;\left(100\%\right)$$

where *ρ_a_* and *ρ_l_* are the density of air (1.225 kg m^−3^) and unfoamed polyamide (PA) (1150 kg m^−3^), respectively. The unfoamed PA volume ($V_{l}$) was calculated as:

(3) $$V_{l}=\frac{\rho_{f}-\rho_{a}}{\rho_{l}-\rho_{a}}\;V_{f}$$

while the gas phase (air) occupies a volume of:

(4) $$V_{a}=P\;V_{f}$$

Each sample was then immersed in an ethanol bath for three days. The volume of ethanol absorbed was calculated by using the weight gained (*W_e_*), density of ethanol (*ρ_e_* = 789 kg m^−3^), and mass of polymer in the foam (*V_l_ ρ_l_*) to give:

(5) $$V_{e}=\frac{W_{e}-V_{l}\;\rho_{l}}{\rho_{e}}\;$$

Finally, the open cell percentage (*OCP*) was obtained from the ratio between the volume of ethanol and air as:

(6) $$OCP=\frac{V_{e}}{V_{a}}\;\;\left(100\%\right)$$

### Characterization—Foam Morphology

The cell size of the Nylon foams was quantified by a scanning electron microscope (SEM) Zeiss Crossbeam 540 FIB/SEM (Carl Zeiss, Germany). The cross‐section of each sample was obtained by cutting 1.5 mm thick slices with a doctor's blade before being gold coated to take images at 1.5 kV. The images were then quantitatively analyzed (morphological analysis) to get the cell diameters via the Image J software (National Institutes of Health, USA).

### Characterization—Differential Scanning Calorimetry (DSC)

Differential scanning calorimetry (DSC) measurements were performed using a DSC 25 (TA Instruments Waters, USA) coupled to the TRIOS software with samples weighting ≈3 mg. The temperature was increased at 10 °C min^−1^ from 50 to 280 °C before cooling back to 50 °C and heating again to 280 °C. All the runs were carried out under a constant rate (50 ml min^−1^) of dry nitrogen. The crystallinity (*X_c_*) was calculated as:

(7) $$X_{c}=\frac{\Delta H_{m}}{\Delta H_{100}}\;\left(100\%\right)$$

where ΔHm is the enthalpy of the sample during the heating process, while $\Delta H_{100}$ is the enthalpy of 100% crystalline Nylon 6 taken as 241 J g^−1^.^[^ ^20^ ^]^

### Characterization—Poisson's Ratio

The Poisson's ratios were determined under tension and compression. The tensile tests were done on a dynamic mechanical analyzer (DMA) RSA 3 (TA Instruments, USA), while the compressive test was made on an Instron 5565 (Instron, USA). The deformation rates were fixed at 1.2 mm min^−1^ and the deformations were determined using a high‐resolution video camera (SONY FDR‐AX53, Japan) in two perpendicular directions. The Poisson's ratio (ʋ) was obtained as the negative ratio between the transverse (ε_y_ or ε_z_) and axial (ε_x_) deformations as:^[^ ^21^ ^]^

(8) $$v=-\frac{\varepsilon_{y}}{\varepsilon_{x}}\;\mathrm{or}-\frac{\varepsilon_{z}}{\varepsilon_{x}}$$

More information on the methodology and calculations can be found in our previous publications.^[^ ^2^, ^3^, ^4^, ^18^ ^]^

### Characterization—Mechanical PROPERTIES

The tension and compression stress‐strain data were obtained by both the RSA 3 and Instron 5565. The tests were also done at 1.2 mm min^−1^ for both tension and compression and the moduli were determined by the slope in the linear region of the stress‐strain curves (below 5% deformation).

The melting temperature (Tm) was determined using a dynamic mechanical thermal analyzer (RSA 3, TA Instruments, USA) by increasing the temperature from 50 to 220 °C at a rate of 10 °C min^−1^ with a strain of 0.2% and a frequency of 1 Hz. The dimensions were ≈3 × 5 × 30 mm.

## Conflict of Interest

The authors declare no conflict of interest.

## Data Availability

The data that support the findings of this study are available from the corresponding author upon reasonable request.
